# Supplementary material for: Genetic architecture of heart mitochondrial proteome influencing cardiac hypertrophy
Source: eLife. 2023 Jun 5;12:e82619. doi: 10.7554/eLife.82619 (PMC10241513; doi:10.7554/eLife.82619)
Supplement: Figure 2—source data 1. — Uncropped immunoblots probed for NDUFS4 (left) and ACTIN (right) protein levels in NRVMs transfected with mature miR-27b in the presence or absence of PE treatment. Corresponding molecular weight markers are labelled on the right side of each blot. [file elife-82619-fig2-data1.pdf]

Figure 2, panel J

Original – uncropped

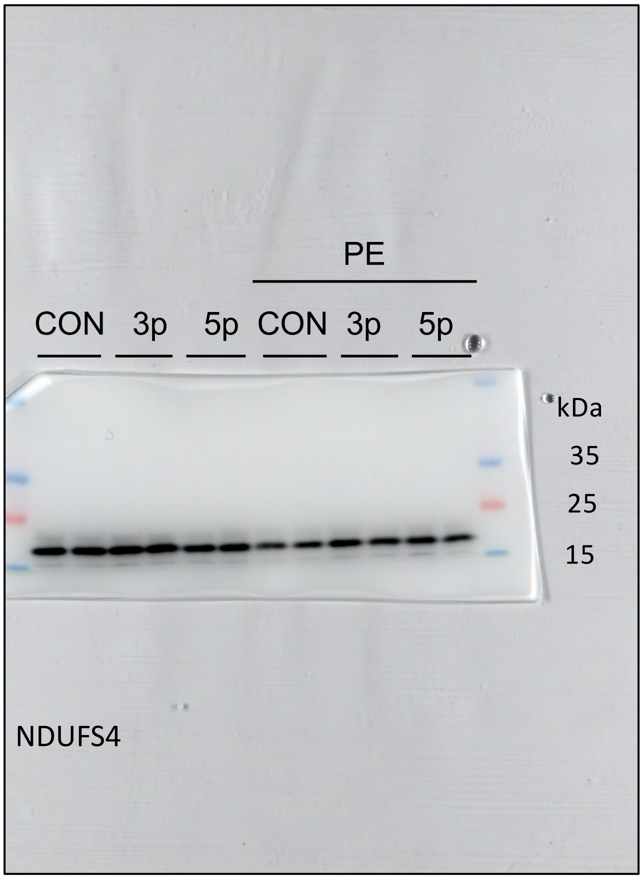

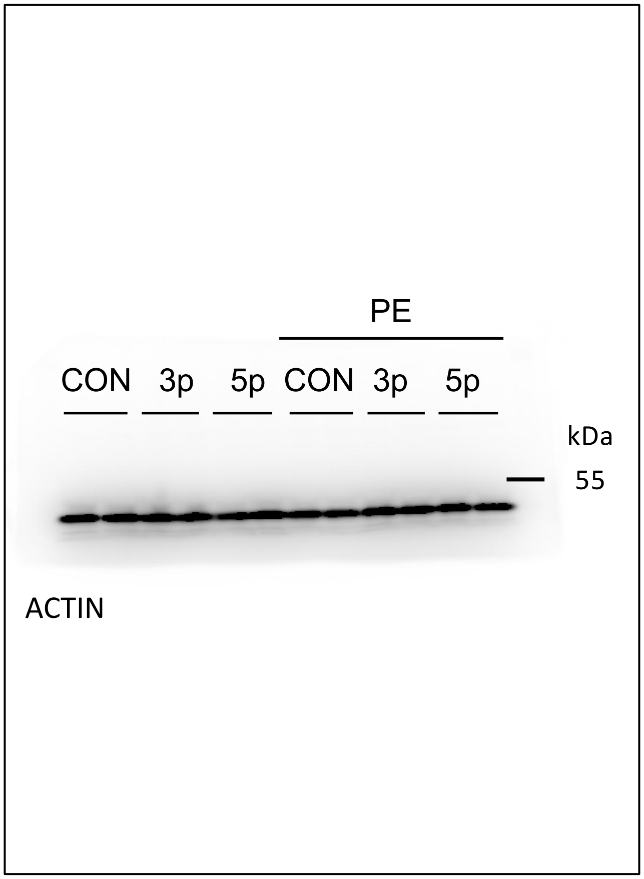

Samples: CON, CON, miR-27b-3p, miR-27b-3p, miR-27b-5p, miR-27b-5p
CON (PE), CON (PE), miR-27b-3p (PE), miR-27b-3p (PE), miR-27b-5p (PE), miR-27b-5p (PE)
