# Supplementary figures and images for: Genetic architecture of heart mitochondrial proteome influencing cardiac hypertrophy
Source: eLife. 2023 Jun 5;12:e82619. doi: 10.7554/eLife.82619 (PMC10241513; doi:10.7554/eLife.82619)

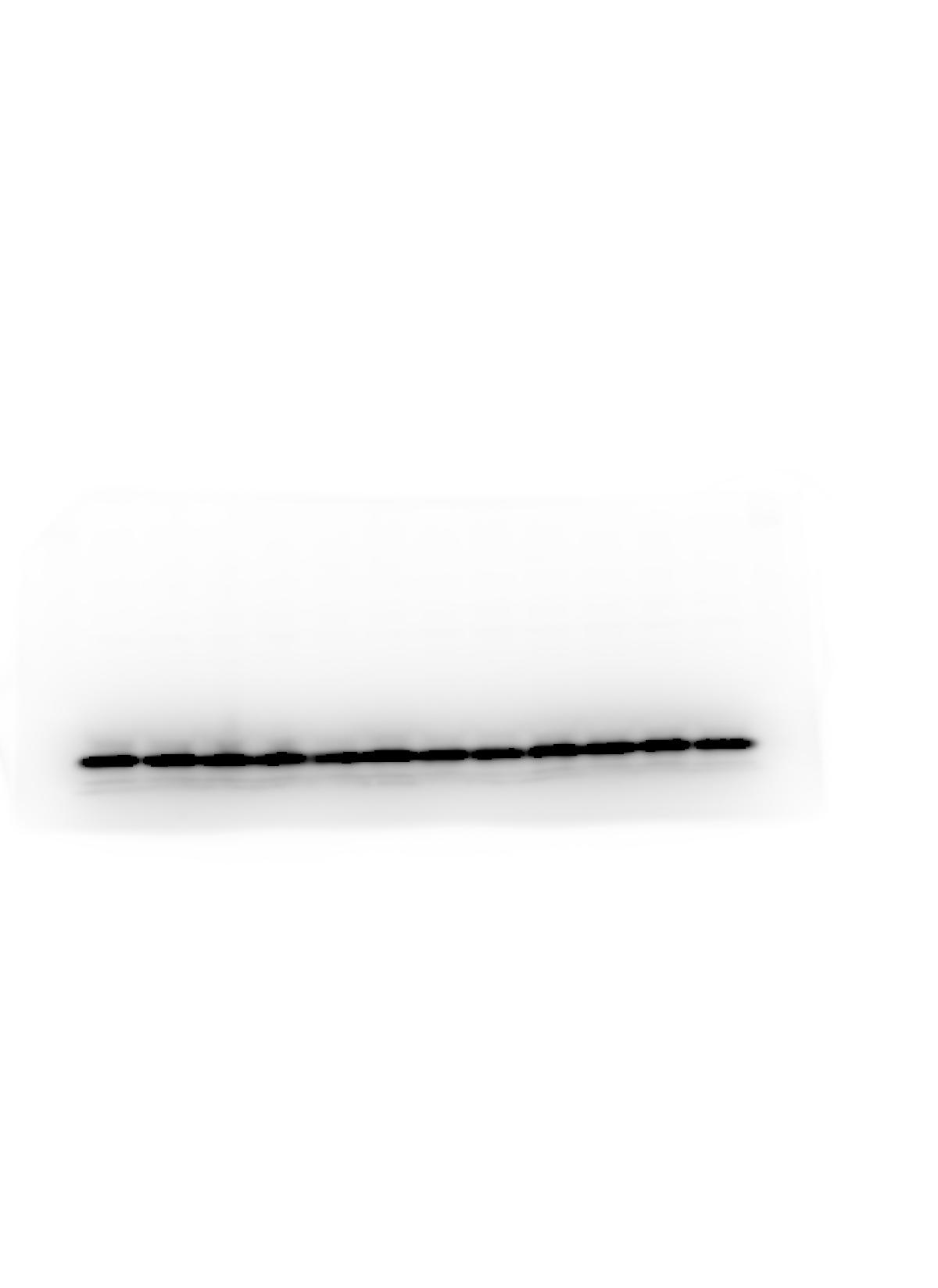

Supplement: Figure 2—source data 2. [file elife-82619-fig2-data2.zip › Figure 2-source data 1/Figure 2-source data 1 - right gel - ACTIN.jpg]

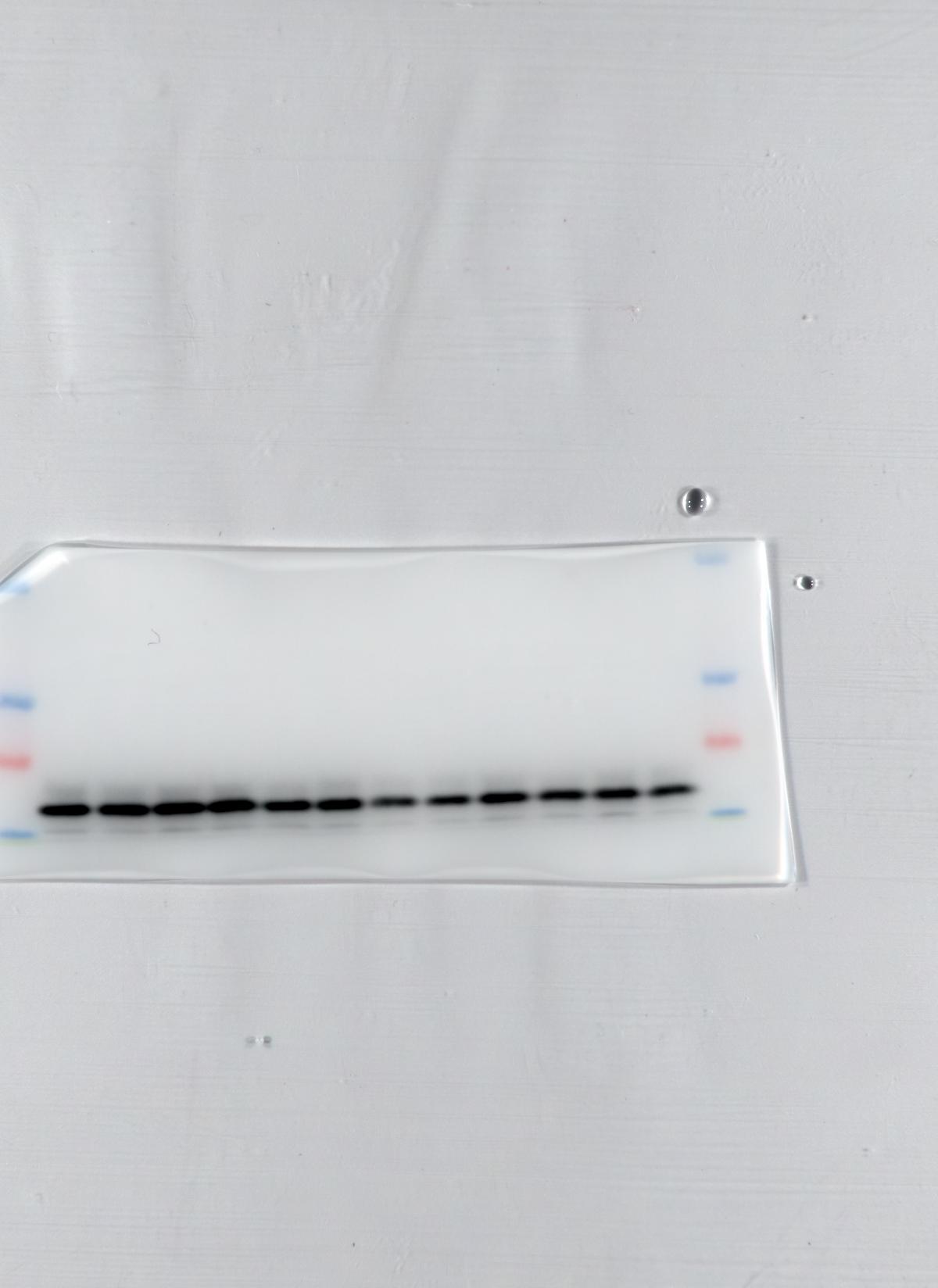

Supplement: Figure 2—source data 2. [file elife-82619-fig2-data2.zip › Figure 2-source data 1/Figure 2-source data 1 - left gel - NDUFS4.jpg]
